# COVID-19 vaccinations in The Gambia: efforts, challenges, and future directions

**DOI:** 10.11604/pamj.2022.42.164.34199

**Published:** 2022-06-30

**Authors:** Mat Lowe, Nicolas Edgar, Don Eliseo Lucero-Prisno

**Affiliations:** 1 Society for the Study of Women’s Health (SSWH), Kanifing, The Gambia,; 2 University of California, Los Angeles, California, United States of America,; 3 Department of Global Health and Development, London School of Hygiene and Tropical Medicine, England, United Kingdom

**Keywords:** Coronavirus, The Gambia, COVID-19, efforts, challenges

## Abstract

On March 11, 2020, the novel coronavirus disease (COVID-19) was declared a pandemic. Since the declaration, countries have implemented response measures to stop the spread of the virus, while multiple vaccines combatting the virus have also been developed. However, vaccine rollout and providing vaccine access has been very challenging in many African countries, including The Gambia. This article briefly assesses the efforts and challenges facing The Gambia´s COVID-19 vaccine rollout and implementation. The article also provides recommendations that policymakers and program implementers can use to address the low COVID-19 vaccination rate in The Gambia. It is based on a narrative review of existing literature on COVID-19 vaccination efforts and challenges in The Gambia.

## Commentary

**COVID-19 vaccination efforts in The Gambia:** the first case of COVID-19 in The Gambia was confirmed on March 17, 2021, and as of February 28, 2022, the total number of COVID-19 cases reached nearly 12,000 with 365 total deaths from the disease [1,2]. An effective distribution of COVID-19 vaccines could mitigate future waves of coronavirus infections, while allowing the country to keep public spaces open amid a return to normal life. COVID-19 vaccines first arrived in The Gambia in March of 2021, when a COVID-19 Vaccines Global Access (COVAX) vaccination rollout was launched. Thirty-six thousand doses of Oxford Astra-Zeneca vaccines arrived under the COVAX facility with the launch. The first contingent of vaccines was prioritized for healthcare workers, people with chronic conditions, and those 65 or older to minimize the severity of the disease if exposure occurred [3]. In April 2021, The Gambia received an $8 million funding grant from the International Development Association for “safe and effective vaccine purchase and deployment” through the Gambia COVID-19 vaccine preparedness and response project [4]. Since then, The Gambia has received an additional 405,100 doses of COVID-19 vaccines, including 376,800 through the COVAX initiative, 52,800 through the African Vaccine Acquisition Trust (AVAT), and 11,500 doses via bilateral agreements [4]. However, only 349,875 doses had been administered as of February 16, with none of the AVAT doses being used [5]. In total, 79% of available vaccines have reached The Gambian population [5], with between 13% and 20% of the population counting as fully vaccinated [2,6].

The Gambia initially proposed goals of vaccinating 40% of its population by December 2021 and implemented mobile vaccinations campaigns in the summer of 2021 which increased vaccination rates in that timeframe. Furthermore, house-to-house outreach campaigns between December 2021 and January 2022 improved the population reached by vaccination campaigns when compared to the fixed-site strategy used since March 2021 when vaccines initially reached The Gambia [7]. In January 2022, the International Organization for Migration (IOM) launched a campaign to vaccinate mobile populations along The Gambia´s border with Senegal [8]. So far, the initiative has vaccinated 1,500 individuals in 60 border communities, with The Gambian, Ministry of Health planning three more similar campaigns in the Central River, Lower River, and West Coast Regions of The Gambia [8]. However, despite these initiatives, the vaccination rate hovers around 20% as of 18^th^ February 2022.

**Challenges of the COVID-19 vaccination rollout in The Gambia:** a major challenge in The Gambia´s vaccination efforts against COVID-19 has been finding a balance between mobile outreach campaigns and fixed-site vaccination options. While outreach campaigns have increased demand transiently, maintaining a consistent level of vaccination rates has proven difficult for The Gambia. Furthermore, while projects such as the IOM campaigns targeting mobile at-risk groups present a step in the right direction, the continued low vaccination rate increases the risk for virus transmission and novel, more-transmissive variant emergence. In addition, although COVID-19 vaccinations are available in The Gambia, challenges such as misconceptions about and lack of trust in COVID-19 vaccines, lack of a robust electronic database to track vaccinated individuals, limited vaccination points and staffing shortages may also be significantly affecting the efficiency of the COVID-19 vaccine rollout in The Gambia [3]. Of these issues, mistrust in COVID-19 vaccines driven by misinformation may be the most important to consider and the most difficult to address [9]. The Africa Centers for Disease Control and Prevention study indicated COVID-19 vaccination mistrust and hesitancy as significant challenges in the COVID-19 vaccination rollout in fifteen African countries [10].

While the study did not detail The Gambia, it studied the neighboring Senegal, finding that 50% of surveyed Senegalese citizens agreed with the statement that the coronavirus threat was exaggerated, while 32% of Senegalese surveyed reported that they would not be willing to receive a COVID-19 vaccine. Furthermore, only 29% of Senegalese respondents strongly agreed that a COVID-19 vaccine was safe, with 23% of respondents strongly disagreeing that a COVID-19 vaccine was effective [10]. While these findings cannot be applied to The Gambia directly, similar sentiments may be felt in The Gambia as well, driving COVID-19 vaccine mistrust and hesitancy. If current vaccination rates continue, it may take nearly three years to vaccinate another 10% of the Gambian population [2].

**Future directions for COVID-19 vaccinations in The Gambia:** a variety of steps are needed to increase the vaccination rates against COVID-19 in The Gambia. For instance, considering The Gambia has only received enough vaccines to protect a small subset of the population, increasing vaccine availability through more shipments and securing more vaccines through initiatives such as COVAX would aid in getting more Gambians vaccinated.

Furthermore, increasing mobile outreach and house-to-house campaigns encouraging vaccination could reinvigorate the rate of COVID-19 vaccination in The Gambia. Following the lead of other African countries with more successful vaccine rollouts could also pay dividends. For example, Ghana has made use of drones to reach communities situated far from distribution centers, while Botswana made use of emergency operation centers to coordinate vaccine transport. Training vaccinators and ensuring that sufficient support staff is present for either fixed-site centers or mobile initiatives would also increase the ability to use available doses efficiently. Fighting vaccine misinformation may also play a pivotal role in increasing The Gambia´s COVID-19 vaccination rate. On this issue, The Gambia could turn to programs in other African countries and try to emulate them. In Ghana, for instance, a misinformation and rumor management task force has been established by the Ghanaian authorities to address false claims on COVID-19. In Senegal, the free call centers aim to provide fact-based information to citizens to alleviate hesitancy. To adequately track vaccinated individuals, a system is needed to digitize data and verify vaccination. As of now, increasing vaccine access and availability through mobile outreach seems to be a successful path to pursue [7,8], while increased shipments of vaccines should benefit the total vaccination rate, with nearly 80% of received doses being used.

**Conclusion:** to mitigate the challenges of COVID-19 vaccine implementation and rollout in The Gambia, the development of a robust digital system to effectively track vaccinated persons is urgently needed. Additionally, increasing mobile outreach campaigns as well as door-to-door vaccination campaigns could benefit dwindling vaccination rates. Ensuring proper staffing and training for medical and allied health professionals on these campaigns will be crucial, as will finding a way to address vaccine hesitancy and mistrust. Lastly, The Gambia has shown an ability to use a high percentage of available doses, suggesting increasing dose availability may help vaccinate more Gambians.
